# Letter from Paris

**Published:** 1890-09

**Authors:** H. E. Hayd

**Affiliations:** Paris


### Correspondence.

### LETTER FROM PARIS.

*Second Letter from Dr. Hayd — He Still Praises Apostoli's Work — Galvano-Puncture Especially Endorsed — Terrier and Doleris are also Doing Good Work — Details of Cases Seen in the Clinics.*

In my last letter, in a general way, I spoke of the work at the Rue du Jour; and now, after having spent six weeks in regular attendance, and with the recent cases fresh in my mind, I can speak more definitely of Apostoli's work. It continues to please me, and the results of galvano-puncture, when properly performed, are very satisfactory. I have seen a good many patients in whom a galvano-puncture was made, and I have never seen any bad effects, nor observed any complication follow it. I never have seen a great unsightly slough, or a deep circuitous suppurating track, as I had been told I should see by the opponents of this practice. When a patient is suffering from painful cellulitis, either in the shape of adhesions around the womb, or ligaments, or a painful and often very tender mass in the posterior cul-de-sac; and when ordinary galvano-cauterization has failed to accomplish what was expected, there a puncture may often be made with much satisfaction; of course, a thorough knowledge of the pathological condition is necessary, and then with reasonable care this little operation is a very simple procedure. Apostoli's needle is a sharp-pointed instrument — preferably a gold tip, but steel will do — fitted into a handle and surrounded by a movable sheath. As much of the point is pushed out of the sheath and exposed as you desire to penetrate into the tissues: usually for parametritic exudations three millimeters is all that is necessary, and five mm. for a fibroid. Thus you can see the punctures are very superficial, and possible danger reduced to a minimum. The sheath is directed along the finger to the painful spot, and, if possible, placed where there is no blood-vessel, and held in position until the needle is pushed into the mass. About twenty-five to thirty ma. positive galvanism are given for a period of five minutes. There is but little pain during the sêance, and the patients are permitted to walk home in a couple of hours. In cases of very acute and exquisitely sensitive spots chloroform is necessary, because many of these patients are not simply nervous, but would really suffer very greatly if not anesthetized. Moreover, as a precaution, they are put to bed and kept there until morning, when they are permitted to go home. The vagina is packed with iodoform gauze, and changed in a few days. But rarely more than two or three punctures are necessary, and the interval between the punctures is from fifteen days to three weeks. When it is evident that a fibroid is not doing as well as is desired under galvano-cauterization, which I assure you is extremely rare, then Apostoli punctures ; fifty and sixty ma. are given and no chloroform is necessary, nor do the patients suffer much pain,—at all events not unsupportable.

I can't help but look upon Apostoli and his work with admiration, particularly in these fields, where we can do so little with the other agencies at our disposition. One case was extremely interesting. A woman of good position came to Apostoli eight years ago with a very large fibroid. She was suffering much pain, and at the same time losing a great amount of blood,—in fact, was a dangerously sick woman. Drs. Pean, Championerre, and other surgeons declined to operate upon her. The abdominal parietes were very thin from distention, and the adhesions were so great that it was impossible to move the tumor laterally. After a few months' treatment her condition improved, the menses came with regularity, and not profuse, the pains were relieved, and the general condition of the woman improved. Gradually the adhesions were absorbed, and to-day, after a period of eight years, we find the tumor still very large, but freely movable, in fact a good case for an operation ; and the abdominal parietes, instead of being thin and tense, are soft, placid, and filled with a great layer of fat. Dr. Apostoli tells me that he has frequently made this operation, and on this account invariably measures the thickness of the external tissues before treatment is begun. Retrocession of the tumor often takes place, while at the same time much fat is developed in the subcutaneous tissues over it. This woman has had no treatment for five years.

A week ago I saw a very interesting case,—in fact, one such as we often see as a complication of an insidious gonorrhea. It enabled me to find out exactly Apostoli's position in reference to the application of electricity in ovariopyo-salpingitis. The patient was a woman twenty-four years of age, and with the history of a sudden and severe illness. Upon examination the abdomen was found to be very tense, painful and tender over the ovaries. Per vaginam, a large mass of cellulitis was felt in the posterior cul-de-sac, extending into the broad ligaments and enveloping the whole womb. The womb was immovable, and the parts very sensitive to the touch. In the right broad ligament there could be felt a distinct globular tumor about the size of a small egg, very firm and not fluctuating. The question at once came up, Was this the tube filled with pus and surrounded by a strong, thick wall of inflammatory exudation, or was it simply a large mass of inflammation which had not advanced so far as to develop a definite cyst ? Here Dr. Apostoli said electricity would direct and confirm him in his diagnosis. He would commence to treat her with twenty ma. positive galvanism, intrauterine, which if borne well would be increased. After four or five treatments, if no pus was present, he would expect the woman to improve ; but if pus existed, she would not tolerate the electricity, and would not be benefited by it. Then there was but one thing to do, as the suspicious diagnosis would have been confirmed,— an abdominal section or drainage per vaginam would be advised. You will observe, therefore, if Apostoli is satisfied that a definite cyst or abscess exists in the tube, an operation is at once advised, and that he only uses electricity in cases of catarrhal salpingitis or pyosalpingitis, when no pus collection has taken place. I was particularly pleased to see him lay down these conclusions, and at once saw his anxiety to place this poor woman under the best treatment possible for her condition. Would that the few operating surgeons who still disregard the beneficial influences of electricity, were as fair with their patients ; then I am sure fewer women would be spayed, and the opprobrium associated with the indiscriminate castration of females would be lessened ! Another general rule Apostoli lays down so as to make one suspicious of the possible presence of pus is this : When a woman, not hysterical or preternaturally sensitive to pain, complains much when twenty or thirty ma. of positive galvanism is given her, be on the lookout for something serious, perhaps pus.

In my last letter I also spoke of the faradic current — current of tension — and I have seen surprising benefit from it, in that class of nervous women who complain of painful iliac regions, tender upon pressure, with dysmenorrhea, and often dyspareunia, and vaginismus. Many of these patients are very sensitive to deep pressure in the epigastric region, and scream when pressure is made in the pit of the stomach.

My admiration for Terrier has increased, and I believe he does the best abdominal work in Paris. Doléris, another man celebrated for his operations in the vagina and upon the perineum, I have also visited quite frequently. Besides a number of Emmet operations which I have seen him make, I was particularly pleased with his modified Tait's operation for lacerated perineum, colpo-perineo-plasty. The case was a marked rectocele, due to a large, soft relaxed vagina, with little or no supporting tissue in the perineum itself. A circular incision is made at the junction of the vaginal mucous membrane and skin, and with the index finger one separates the vaginal mucous membrane posteriorly and laterally for a good distance, and as high up, if necessary, as the cervix uteri. Then the perineal edges are brought together with three or four deep sutures made of florence gut. This is a very nice ligature and very strong, and is made from the silkworm's gut. After the perineal edges are approximated, the vaginal orifice is much reduced in size, and the little piece of loose superfluous mucous membrane which protrudes through the orifice is snipped off and the free edge picked up with a suture. This operation is employed for all lacerations of the perineum, and is modified somewhat for prolapsed bladder. There is very little hemorrhage, the operation is very quickly done, and there is left in the vagina for its posterior wall the natural vaginal tissue, instead of the scar tissue, which results from an ordinary perineorrhaphy.

Silk ligatures are used altogether by French surgeons in the abdomen, and often are very, very large. The pedicle of an ovarian cyst or base of a tube case are tied with a ligature as thick as our ordinary grocer's twine. The superficial skin ligatures are of this florence gut. There is no feeling of hesitancy in applying any number of silk sutures in a wound of any description.

To avoid shock during and after an operation, the patient's extremities are wrapped in flannel to the thighs to keep them warm. Chloroform is invariably used in all the hospitals — in fact, I have never seen ether given.

H. E. HAYD.

Paris, July 4, 1890.

---

An absent-minded doctor recently took unto himself a wife. During the marriage ceremony, when she held out her hand for the ring, he felt her pulse and requested to see her tongue.

